# *ACTB* Mutations Analysis and Genotype–Phenotype Correlation in Becker’s Nevus

**DOI:** 10.3390/biomedicines9121879

**Published:** 2021-12-10

**Authors:** Shangzhi Dai, Huijun Wang, Zhimiao Lin

**Affiliations:** 1 Department of Dermatology, Peking University First Hospital, Beijing Key Laboratory of Molecular Diagnosis on Dermatoses, National Clinical Research Center for Skin and Immune Diseases, Beijing 100034, China; szdai@bjmu.edu.cn (S.D.); whj19860216@126.com (H.W.); 2 Dermatology Hospital, Southern Medical University, Guangzhou 510091, China

**Keywords:** Becker’s nevus, mosaicism, *ACTB* mutation, genotype–phenotype correlation

## Abstract

Becker’s nevus (BN) is a cutaneous hamartoma which is characterized by circumscribed hyperpigmentation with hypertrichosis. Recent studies have revealed that BN patients harbored postzygotic *ACTB* mutations, which were restricted to arrector pili muscle lineage. We screened for *ACTB* mutations in 20 Chinese patients with BN and found that recurrent mutations (c.C439A or c.C439T) in *ACTB* were detected in the majority of BN patients. However, more than 20% of the patients were negative for *ACTB* mutations, suggesting a possible genetic heterogeneity in Becker’s nevus. Interestingly, these mutations were also detected in dermal tissues outside the arrector pili muscle. We further performed genotype–phenotype correlation analysis, which revealed that lesions above the waistline, including the trunk above the anterior superior spine level, upper limbs and face, or covering more than 1% BSA were more likely to be positive for *ACTB* mutations. Altogether, our results provide further evidence of postzygotic *ACTB* mutations in BN patients and suggest a possible genotype–phenotype correlation of BN.

## 1. Introduction

Becker’s nevus (BN), also known as pigmentary hairy epidermal nevus, is a relatively common disease. The prevalence is estimated at around 0.25–2%, and it varies with ethnicity and region [1,2]. Skin lesions mostly appear after puberty, characterized by unilateral, brown-black pigmented patches with or without hypertrichosis. The predilected sites for BN are chest and upper limbs [3,4,5]. Facial involvement is relatively rare but might give rise to a considerable cosmetic concern [6]. Recurrent acne and eczema in the affected areas were occasionally reported [7,8]. Becker’s nevus syndrome (BNS) refers to conditions in which generalized BN is associated with developmental anomalies, including unilateral breast hypoplasia and other cutaneous, muscular, and skeletal defects. Histopathologically, BN shows hyperkeratosis, acanthosis, regular elongation of the rete ridges and basal hyperpigmentation. Hypertrophy of the arrector pili muscle is commonly seen [3,9].

Previously, BN was established in the literature as an organoid harmatoma and the unilateral distribution might reflect mosaicism [3]. The distinct phenotype of BN and BNS might be explained by mutations at different developmental stages. BN was also considered to be androgen-dependent, given that most of the lesions appeared or became exacerbated during puberty, and there was an increase in expression and sensitivity of androgen receptors in different tissues in BN samples [10].

Recently, patients with BN were found to harbor postzygotic mutations c.C439A (p.R147S) or c.C439T (p.R147C) in *ACTB*, which encodes a ubiquitously expressed protein β-actin. The mutation was detected in the hypertrophic arrector pili muscle in a single BN patient [11]. Herein, we provide further evidence for postzygotic *ACTB* mutations in BN lesions in a multi-lineage background. We also try to investigate the possible genotype–phenotype correlation of BN.

## 2. Materials and Methods

### 2.1. Patients

The medical files and histopathological samples of patients diagnosed with BN in the past two years in Peking University First Hospital were reviewed. Cases diagnosed as Becker’s nevus or Becker’s nevus syndrome were enrolled. The diagnoses were confirmed both clinically and histopathologically by two senior attendings. The study was approved by the Clinical Research Ethics Committee of Peking University First Hospital, Beijing, China (institutional review board number 2016[1024], approval date 28 September 2016). A total of 20 patients with Becker’s nevus, including one with Becker’s nevus syndrome, were selected. The clinical and pathological data were collected. Apart from baseline demographic information such as sex and age of onset, lesion size, location and hairy presentation were examined and recorded. Size of the lesion was measured by percentage of body surface area (BSA), estimated via palm size, each representing 1% BSA. Lesions less than one palm size were assigned to the <1% group. Histological features including hyperkeratosis, elongation/fusion/flatten of the epidermal rete ridge, acanthosis, fibrosis of the dermis, hyperplasia of the arrector pili muscle and sebaceous gland were summarized.

### 2.2. Genomic DNA Extraction

Genomic DNA was extracted from FFPE sections or tissue isolated by laser capture microdissection (LCM). DNA extraction of FFPE samples was performed using the QIAamp DNA FFPE Tissue Kit (QIAGEN, Hilden, Germany), according to the manufacturer’s protocol, with a prolonged digestion time of 24 h. For LCM samples, QIAamp DNA Micro Kit (QIAGEN, Hilden, Germany) was used.

### 2.3. Polymerase Chain Reaction and Sequencing

**Figure 1:**
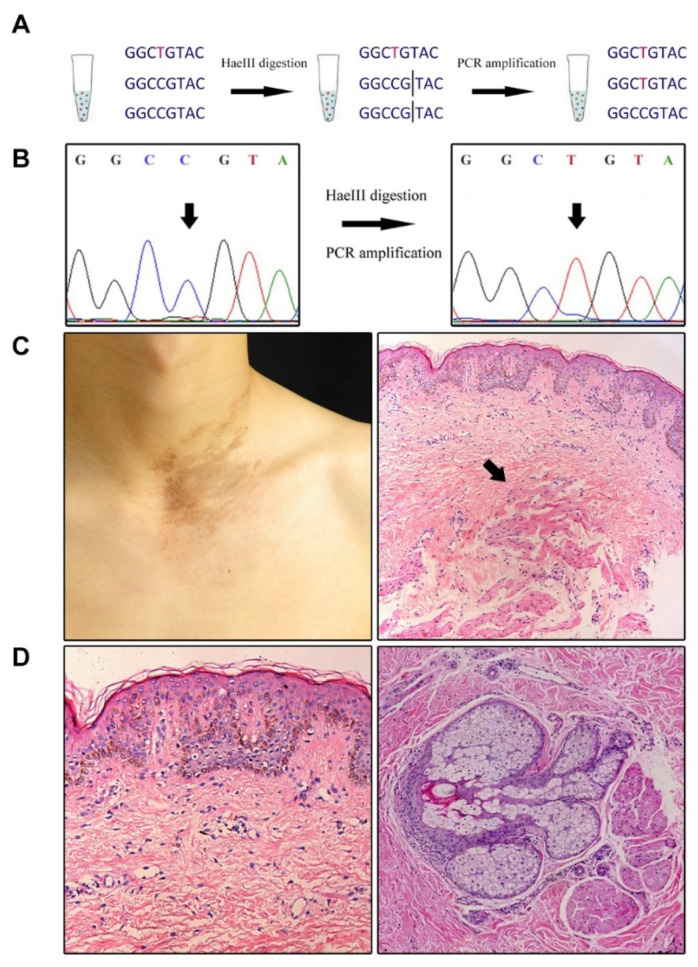
Patient samples and methods of *ACTB* mutation detection. (**A**) Workflow of the enrichment assay. (**B**) An example showing that the proportion of mutated allele was increased after enrichment process. (**C**) Clinical (left panel) and pathological (right panel, original magnification ×20) features of one Becker’s nevus patient, the arrow indicating the increased arrector pili muscle. (**D**) Characteristic pathological changes of Becker’s nevus showing basal hyperpigmentation, epidermal rete ridge elongation, fusion and flatten, dermal lymphocyte infiltration and fibrosis (left panel, original magnification ×40), sebaceous gland and arrector pili muscle hyperplasia (right panel, original magnification ×40).

DNA extracted from FFPE or LCM samples was amplified, and the amplicons were subjected to HaeIII digestion for 180 min to enrich mutated alleles. The digested products were purified using the QIAquick PCR Purification Kit (QIAGEN, Hilden, Germany) and subjected to a second round of PCR amplification (Figure 1A,B). Primer sequences are shown in Table S1. The PCR products were Sanger sequenced [11].

### 2.4. Statistics

SPSS edition 23 (IBM, Armonk, NY, USA) software was used for statistical analysis. Patients were separated into two groups based on their clinical features and whether or not they harbored *ACTB* mutations. A chi-square test was utilized to determine if patients with certain features were more likely to carry corresponding *ACTB* mutations. The level of statistical significance was determined to *p* values < 0.05.

## 3. Results

### 3.1. ACTB Mutations Screening and Histological Location

To screen for c.C439A or c.C439T mutation in *ACTB* in selected patients, DNA was extracted from whole paraffin samples and subjected to HaeIII digestion and nest PCR as previously described. Among the 20 patients enrolled, 15 (75%) were found to have *ACTB* mutations. Fourteen patients harbored c.C439T mutations, and one had c.C439A mutation (Table 1). Five patients, including the syndromic case, were not detected with *ACTB* c.C439A or c.C439T mutations. For the patient with BNS, DNA extracted from arrector pili muscle tissues was amplified and subjected to Sanger sequencing of the whole exonic region of the *ACTB* gene. However, no mutations were found.

**Figure 2:**
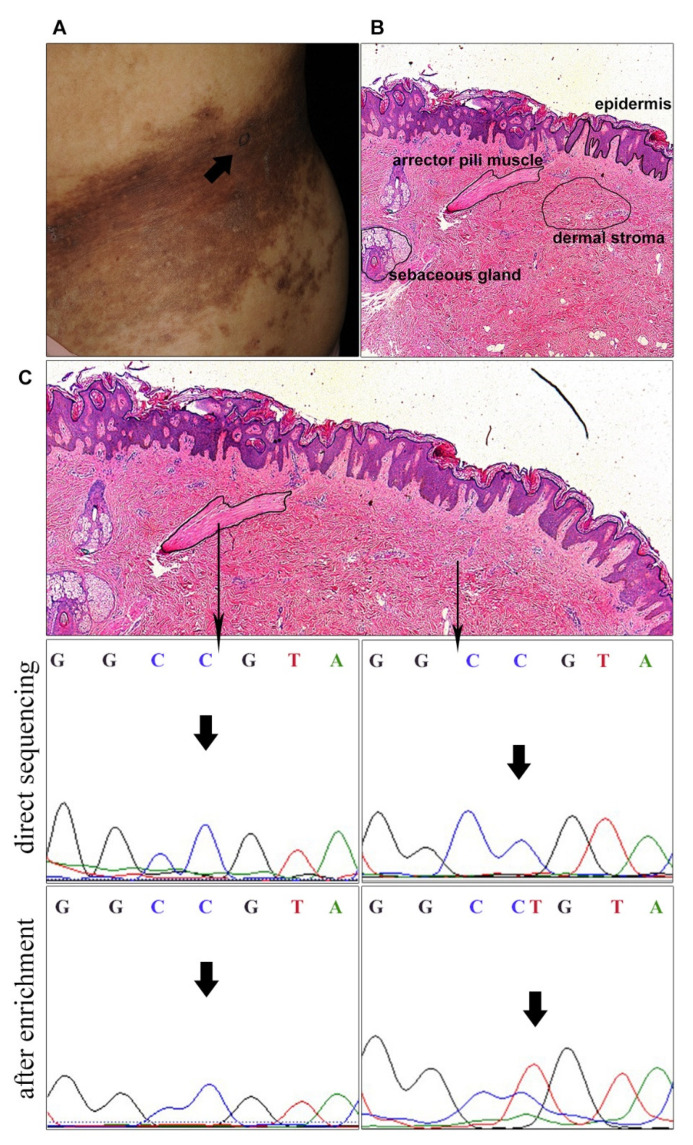
Laser capture microdissection site and results of Sanger sequencing. (**A**) Clinical picture of the patient (No. 1767) with Becker’s nevus, the black arrow indicating the site of skin biopsy. Sample was subjected to LCM shown in (**B**,**C**). (**B**) Epidermis, dermal stroma, hair follicles (not shown), arrector pili muscle and sebaceous gland were isolated. Original magnification ×20. (**C**) DNA was extracted from arrector pili muscle and the remaining tissue. *ACTB* mutations were negative in arrector pili muscle after HaeIII digestion and PCR amplification (left panel), but were detected in the remaining tissue (right panel). The blacks arrow in Sanger sequencing indicate the mutation sites.

In order to locate the *ACTB* mutations at histological level, LCM was performed [11]. Samples from three patients harboring mutation c.C439T in *ACTB,* with hypertrophic arrector pili muscle, were selected (Figure 1C,D). Several tissues that likely had androgen receptor overexpression, according to the previous study, were isolated, including epidermis, dermal stroma, hair follicles, arrector pili muscle and sebaceous glands (Figure 2A,B) [10]. Extracted DNA was PCR amplified and Sanger sequenced. However, no mutations were found in any of these tissues.

*ACTB* mutations were reported to be restricted to the arrector pili muscle [11]. We suspected that mutation ratios might be too low for direct Sanger screening in these isolated tissues. Therefore, we further performed the enrichment assay, utilizing HaeIII enzymatic digestion on DNA from the arrector pili muscle. The targeted region was negative for c.C439T *ACTB* mutation. We removed arrector pili muscle using LCM and extracted DNA from the remaining tissue. After two rounds of PCR amplification and HaeIII enzymatic digestion, Sanger sequencing revealed the mutation c.C439T in *ACTB* (Figure 2C). This suggests the possible existence of an additional mutation lineage of *ACTB,* besides the arrector pili muscle.

### 3.2. Clinical and Histological Information of the Patients

We enrolled 20 patients with BN, including 1 patient with Becker’s nevus syndrome. Considering the generalized involvement and distinct phenotype, the syndromic patient was excluded from clinical and pathological analyses. The demographic analysis showed a male predominance of 14:5 in sex ratio (Table 1). Most of the lesions appeared during puberty or thereafter (>10 years of age, 12 cases, 63.2%). Lesions were mostly located above the waist (14 cases, 73.7%), which include the trunk (above the anterior superior spine level), upper limbs and face. More than 50% of the lesions were located on the limbs (11 cases, 57.9%). Regarding the inaccuracy of palm size estimation, lesion sizes were divided roughly into two groups by percentage of BSA: <1% (six cases, 31.6%) and ≥1% (13 cases, 68.4%). Twelve cases (63.2%) presented with hypertrichosis in the affected skin. Detailed clinical information is shown in Table S2.

Histopathological samples were reviewed. All the cases presented with epidermal rete elongation and lymphocytes infiltration in the dermis. The other common manifestations include fusion (18 cases, 94.7%) and flattening (18 cases, 94.7%) of the epidermal rete ridges, hyperkeratosis (11 cases, 57.9%), acanthosis (12 cases, 63.2%), hyperpigmentation in basal layer (15 cases, 78.9%) and hyperplasia of the arrector pili muscle (13 cases, 68.4%). Features including hyperplasia of the sebaceous glands (four cases, 21.0%) and fibrosis of dermis (seven cases, 36.8%) were also observed. Histological data are summarized in Table S3.

### 3.3. Genotype–Phenotype Correlation Analysis

We performed an analysis of the correlations of clinical and pathological presentations with *ACTB* mutations. No statistical significance was achieved in either sex (*p* = 1.000) or age of onset (*p* = 0.603) in both groups (with or without mutations in *ACTB*). A positive correlation between lesions sizes (*p* = 0.004), location (*p* = 0.037) and *ACTB* mutations was observed. Patients with lesions above the waistline, including on the trunk above the anterior superior spine level, upper limbs and face or covering more than 1% BSA were more likely to harbor *ACTB* mutations. No significant correlation with *ACTB* mutation was observed in patients with skin lesions on limbs or the axial region (torso and head). Pathological features including keratotic hyperplasia, acanthosis, hyperpigmentation of basal layer, hyperplasia of sebaceous gland and arrector pili muscle hypertrophy were analyzed. The results indicated no statistical significance, suggesting no correlation in patients with *ACTB* mutations regarding pathological phenotypes. Results of the analysis of the correlations of clinical and pathological presentations with *ACTB* mutations are shown in Table 2 and Table 3.

## 4. Discussion

BN and BNS are typical examples of mosaic disorders [3]. Postzygotic mutations in *ACTB* underlying BN have been reported recently [11]. Our study provides further evidence of postzygotic *ACTB* mutations in the pathogenesis of BN patients. According to the previous results, *ACTB* mutations were reported to be located in the hypertrophic arrector pili muscle. Interestingly, we found *ACTB* mutations in dermal tissues other than the non-arrector pili muscle, implying that the arrector pili muscle might be not the only origin of *ACTB* mutations in BN. We postulated that *ACTB* mutations can also be located in cell lineage that were of small fraction, such as fibroblast. Considering the fact that specific lineages of fibroblast in the upper dermis contribute to the development of dermal papilla and arrector pili muscle, mutations in fibroblast have the potential to cause multi-lineage involvement [12]. Recently, *ACTB* mutations were detected in cultured fibroblast from patients with congenital smooth muscle hamartoma [13]. Given the overlap of the phenotypes between BN and congenital smooth muscle hamartoma, there might be shared pathogenesis of these two entities. Further study on isolated fibroblast from skin lesions of the BN patients is warranted to determine possible mosaic constellations within the lineage. Mutations at different developmental stages involving varied cell lineages can explain the different clinical presentations of diseases caused by *ACTB* mutations, including Baraitser–Winter syndrome, and juvenile onset dystonia, both of which cause severe neural and musculoskeletal defects [14,15]. The fact that mutations in one single-cell lineage can cause multi-lineage involvement may provide novel clues to understand inter-lineage crosstalk during skin development.

Interestingly, we unraveled a possible genotype–phenotype correlation which showed that patients with skin lesions located above the waist or covering more than 1% BSA were more likely to harbor *ACTB* mutations, as shown in Table 2. Regarding the genetic heterogeneity of Becker’s nevus, lesions on different locations may result from mutations at different hotspots or in different genes. One example is that in patients with port-wine stain, lesions on the face and limbs have distinct genetic backgrounds, which suggests there can be an unknown pathogenesis of Becker’s nevus located below the waist [16].

One explanation for the correlation of lesion size and genetic mutations is that larger lesions have an increased proportion of mutated cells, which is more likely to be detected via enrichment assay. Previously, deep sequencing has been applied in the detection of mutations in mosaic skin disorders. For lesions without *ACTB* mutations, deep sequencing can be an alternative option. Besides, those lesions covering less than 1% of BSA might also harbor mutations at different sites in *ACTB*. Different lesion sizes might be the reflection of the proliferation capacity of cells mutated at different hotspots in *ACTB*. For those not detected with *ACTB* c.C439A or c.C439T mutations, next-generation sequencing (NGS) targeting the entire *ACTB* gene can be performed. In two patients with lesions on the lower limbs, c.C439T mutations in *ACTB* were detected, which might be explained by their lesion sizes (>19% and 3% BSA, respectively).

No statistical significance was achieved on lesions of limbs or axial distribution in patients with or without *ACTB* mutations. However, all of the mutation-negative lesions were located on the limbs. As all of the lesions on the limbs were less than 1% BSA and not detected with *ACTB* mutations, lesion size might be a confounding factor in the correlation between *ACTB* mutations and locations of the lesion.

No significant correlation between histopathological features and *ACTB* mutations was observed, which might be due to the multi-lineage nature of mutated cells in Becker’s nevus (Table 3). The multi-lineage involvement of histopathological changes can also be the results of mutations in cells with stemness, of which the mutation at different stages could lead to varied histopathological manifestations, thus blurring the clues of genotype–phenotype correlation. However, the possible correlation could not be ruled out, since histopathological specimens are discrete images which may not reflect the whole histological changes. Due to a limited sample size, the correlation between histopathological manifestations and genotype in our study might be inconclusive. Further study based on a larger scale is warranted.

Together, our data further supported the pathogenic role of *ACTB* mutation in BN and suggested other possible histological location of the mutations. We also proposed a possible genotype–phenotype correlation in Becker’s nevus which showed that lesion size and location can be predictive for *ACTB* mutations. Further studies are warranted to determine the cell-lineage origin and functional changes in the development of Becker’s nevus.

## Figures and Tables

**Table 1:** Demographic and genotype information of Becker’s nevus patients.

Number	Sex	Age of Onset	*ACTB* Nucleic Acid Change	AA Change
2948	F	10	c.C439T	R147C
3138	M	15	c.C439T	R147C
3989	M	0	c.C439T	R147C
4108	F	10	c.C439T	R147C
4066	F	30	WT	WT
4068	M	0	WT	WT
4229	M	66	c.C439A	R147S
4305	M	20	WT	WT
4377	F	0	c.C439T	R147C
4448	M	16	c.C439T	R147C
0591	M	0	c.C439T	R147C
1624	M	13	c.C439T	R147C
1767	F	22	c.C439T	R147C
0190	M	15	c.C439T	R147C
0198	M	11	c.C439T	R147C
0497	M	0	c.C439T	R147C
3199	M	1	c.C439T	R147C
0738	M	11	c.C439T	R147C
0051	M	0	WT	WT
CHT01 *	F	8	WT	WT
Mean	/	12.4	/	/

* The patient with Becker’s nevus syndrome. WT, wildtype; M, male; F, female;/, not applied.

**Table 2:** Correlation analysis of clinical data and genotype.

Variable	*ACTB* Mutation Positive (*n*)		*ACTB* Mutation-Positive Percentage	*p* Value
	Yes	No		
Male	11	3	78.6%	1.000
Female	4	1	80.0%	
Birth	5	2	71.4%	0.603
Puberty or after	10	2	83.3%	
Waist and above	13	1	92.9%	0.037
Below the waist	2	3	40.0%	
Torso and head	8	0	100.0%	0.1032
Limbs	7	4	63.6%	
<1% BSA	2	4	33.3%	0.004
≥1% BSA	13	0	100.0%	
With hair	9	3	75.0%	1.000
No hair	6	1	85.7%	

**Table 3:** Correlation analysis of pathological features and genotypes.

Variable	*ACTB* Mutation Positive (*n*)		*ACTB* Mutation-Positive Percentage	*p* Value
	Yes	No		
Hyperkeratosis				0.262
Yes	10	1	90.9%	
No	5	3	62.5%	
Acanthosis				0.117
Yes	11	1	91.7%	
No	4	3	57.1%	
Basal hyperpigmentation				0.178
Yes	13	2	86.7%	
No	2	2	50.0%	
Sebaceous gland hyperplasia				1.000
Yes	3	1	75.0%	
No	12	3	80.0%	
Hyperplasia of arrector pili muscle				0.071
Yes	12	1	92.3%	
No	3	3	50.0%	

## Data Availability

Raw data are available from the corresponding author upon request.

## Supplementary Materials

The following are available online at https://www.mdpi.com/article/10.3390/biomedicines9121879/s1, Table S1: Primer sequence of *ACTB* gene. Table S2: Clinical information of the patients with Becker’s nevus. Table S3: Pathological data of the patients with Becker’s nevus.
