# Supplementary material for: ACTB Mutations Analysis and Genotype–Phenotype Correlation in Becker’s Nevus
Source: Biomedicines. 2021 Dec 10;9(12):1879. doi: 10.3390/biomedicines9121879 (PMC8698930; doi:10.3390/biomedicines9121879)
Supplement: Supplementary file 1 [file biomedicines-09-01879-s001.zip › biomedicines-1458719-supplementary.pdf]

Table S1. Primer sequence of *ACTB* gene.

Number	Sequence
Primer-Exon 1	
Forward	GACCTCGGCTCACAGCG
Reverse	CCTGTGCAGAGAAAGCGCC
Primer-Exon 2	
I-Forward	AGGGCTTCTTGTCCTTTCCTTCC
I-Reverse	TTGTAGAAGGTGTGGTGCCAGATTT
II-Forward	CATCGAGCACGGCATCGT
II-Reverse	GAAAACGGCAGAAGAGAGAACCA
Primer-Exon 3	
I-Forward	TTCTGCATGTCCCCCGTCT
I-Reverse	GGAGGGCATACCCCTCGTAGA
II-Forward	GTGATGGACTCCGGTGACG
II-Reverse	GGTAACCCTCATGTCAGGCAGA
Primer-Exon 4	
Forward	TGTGGAAGCTAAGTCCTGCC
Reverse	GTGACAGCTCCCCACACAC
Primer-Exon 5	
Forward	GAGCTGTCACATCCAGGGTC
Reverse	TGCGCAAGTTAGGTTTTGTCA
Primer-before digestion*	
Forward	ACATGGTGTATCTCTGCCTTACA
Reverse	GAGGGCATACCCCTCGTAGA
Primer-after digestion*	
Forward	CTTCAACACCCCAGCCATGT
Reverse	TAGATGGGCACAGTGTGGGT

* Primers used in the enrichment assay.

Table S2. Clinical information of the patients with Becker's nevus.

Number	location	Size	Hair	Other
2948	left shoulder	2%	√	Papules, scales
3138	right arm	1%	√	Papules, erythema
3989	right shoulder	3%	√	Papules
4108	right chest	1%	×	Papules
4066	left knee	＜1%	√	Erythema
4068	right ankle	＜1%	×	Scales
4229	right arm	＜1%	√	
4305	right elbow	＜1%	√	Erythema
4377	right leg	19%	√	
4448	right shank	3%	√	Papules
0591	right face	1%	×	Papules
1624	right waist	1%	√	
1767	left waist	4%	×	Scales
0190	left chest	2%	√	Folliculitis
0198	left chest	1%	×	Papules
0497	back	10%	×	
3199	left neck	＜1%	√	Scales
0738	left shoulder	3%	×	Papules, erythema
0051	left ankle	＜1%	√	

√, with hairy presentation; ×, without hairy presentation.

Table S3. Pathological data of the patients with Becker's nevus.

Number	Epidermal rete			Hyperkeratosis	Acanthosis	Basal hyperpigmentation	Dermal lymphocytes infiltration	Dermal fibrosis	Sebaceous gland hyperplasia	Hyperplasisa of arrector pili muscle
	Elongation	Fusion	Flattening							
2948	√	√	√	√	√	×	√	×	×	√
3138	√	√	√	√	√	√	√	√	×	√
3989	√	√	√	√	√	√	√	×	×	√
4108	√	√	√	√	√	√	√	√	×	√
4066	√	×	×	×	×	×	√	√	×	×
4068	√	√	√	×	×	√	√	×	×	×
4229	√	√	√	√	√	√	√	√	×	×
4305	√	√	√	√	√	×	√	√	√	√
4377	√	√	√	×	×	√	√	×	×	√
4448	√	√	√	√	√	√	√	×	×	×
0591	√	√	√	√	√	√	√	×	×	√
1624	√	√	√	×	×	√	√	×	×	√
1767	√	√	√	√	√	√	√	×	√	√
0190	√	√	√	×	×	√	√	√	√	√
0198	√	√	√	×	√	√	√	×	×	√
0497	√	√	√	√	√	√	√	√	×	×
3199	√	√	√	×	×	√	√	×	×	√
0738	√	√	√	√	√	×	√	×	√	√
0051	√	√	√	×	×	√	√	×	×	×

√, with corresponding presentation; ×, without corresponding presentation.
